# Prevalence of peripheral blood parasitaemia, anaemia and low birthweight among pregnant women in a suburban area in coastal Ghana

**DOI:** 10.11694/pamj.supp.2014.17.1.3541

**Published:** 2014-01-18

**Authors:** Judith Koryo Stephens, Michael F Ofori, Isabella Akyinbah Quakyi, Mark Lee Wilson, Bartholomew Dicky Akanmori

**Affiliations:** 1 Biological, Environmental and Occupational Health Sciences Department, School of Public Health, College of Health Sciences, University of Ghana, P. O. BOX LG 13 Legon, Accra, Ghana; 2 Immunology Department Noguchi Memorial Institute for Medical Research, College of H College of Health Sciences, University of Ghana, P. O. BOX LG 581 Legon, Accra, Ghana; 3 Department of Epidemiology, School of Public Health II, The University of Michigan, 109 Observatory Street, M5507, Ann Arbor, MI 48109-2029, USA; 4 Vaccine Research and Development, Immunization and Vaccines Development Cluster, Office of the Regional Director, WHO regional Office for Africa, Box 06 Djoue, Brazzaville, Congo

**Keywords:** Malaria prevalence, pregnancy, anaemia, birthweight, ITNs, IPTp

## Abstract

**Introduction:**

Malaria and anaemia have adverse effects in pregnant women and on the birth weight of infants in malaria endemic areas. *P. falciparum* malaria, the most virulent species continues to be a major health problem in sub-Saharan Africa. This study was carried out to establish the prevalence of pregnancy-associated malaria and its associated consequences including maternal anaemia and low birthweight (LBW) deliveries and placental malaria among pregnant women in a sub-urban area in coastal Ghana.

**Methods:**

A facility-based investigation was carried out among 320 pregnant women seeking antenatal care in a hospital in suburban coastal Ghana. Information on the use of Insecticide Treated Nets (ITNs) and Intermittent Preventive Treatment in pregnancy (IPTp) were collected using a structured questionnaire at enrolment. Venous blood was collected for microscopy and screening for Glucose 6-phosphate dehydrogenase (G6PD) deficiency. Haemoglobin concentration was obtained from an automatic blood analyzer. Placental smears and birth weight measurements were taken at delivery.

**Resuls:**

The prevalence of *Plasmodium falciparum parasitaemia* was 5%. The mean haemoglobin (Hb) level at registration was 11.44g/dL (95% CI 11.29 – 11.80). Placental blood parasitaemia and low birthweight were 2.5% and 3% respectively. ITN possession was 31.6% with 5.4% usage. The IPTp coverage was 55%.

**Conclusion:**

The prevalence of malaria and anaemia among the pregnant women were low at enrolment. Placental blood parasitaemia and LBW at delivery were also low. These are clear indications of the high coverage of the IPTp. Increase in ITN use will further improve birthweight outcomes and reduce placental malaria.

## Introduction

Malaria, which is transmitted by female anopheles mosquitoes, results in peripheral blood parasitaemia which may be asymptomatic in adults living in malaria endemic areas. Among pregnant women, however, peripheral blood parasites may accumulate in the placenta resulting in malaria-associated pregnancy (PAM) with adverse consequences such as maternal anaemia and low birthweight infants [1]. Interventions such as Intermittent Preventive Treatment in pregnancy (IPTp) and Insecticide Treated Nets (ITNs) prevent PAM by eliminating peripheral parasitaemia or minimizing the exposure to the vector.

Malaria is the main determinant of anaemia among women who are pregnant for the first time in endemic regions [2] whereas iron deficiency contributes more to anaemia among pregnant women who have been pregnant before and are semi-immune to placental malaria. A haemoglobin (Hb) concentration below 11.0g/dl or packed cell volume (PVC) of less than 33.0% is regarded as anaemia [3]. A low Hb has also been identified as a clinical indicator of placental infection with malaria parasites [4]. A significant reduction in mean Haemocrit (Hct) and Hb levels and a higher incidence of anaemia have also been observed among afebrile pregnant women with malaria parasitaemia [2, 5]. It is estimated that about 400,000 pregnant women living in malaria endemic areas in sub-Saharan Africa are at risk for developing severe anaemia (Hb<5.0g/dL) from infection with malaria [6].

Low birthweight (LBW) is a significant risk attributable to malaria in pregnancy. It is estimated that in malaria endemic areas 19% of infant LBW is due to malaria and also that 6% of infant deaths are due to LBW caused by malaria. These estimates imply that around 100,000 infant deaths each year could be due to LBW caused by malaria during pregnancy in malaria endemic areas in Africa [7].The infants of primigravidae are at greatest risk, especially those of older primigravidae [8].

Most studies on the risk of anaemia in pregnancy have examined associations with micronutrient deficiencies [9, 10] or with parity, age, urban/rural disparities and hookworm infection [11]. The present study examined the association between malaria parasitaemia and maternal anaemia and the incidence placental blood parasitaemia and low birth weight of infants among a group of pregnant women making their initial visit for antenatal attendance at a suburban hospital in a low malaria transmission setting in coastal Ghana.

## Methods

### Study setting

The study was conducted at the Alpha Medical Centre (AMC) in Madina town. Madina is situated in the Ga-East District of the Greater Accra Region about 13.5 kilometres from Accra, the capital of Ghana. It is densely populated with an estimated 2007 population of 112,888 from the 2000 census report [12]. The District experiences two distinct rainy seasons from April to June and from September to November with average temperature of about 26°C. Malaria transmission is low to moderate and perennial with considerable seasonal variations and peaks after the rains, between May-October. It is estimated that there are an estimated 20 infective bites per person year [13]. *Plasmodium falciparum* accounts for 98% of all infections reported in the hospital.

### Sample

The number of pregnancies expected in the study area was 20,000 within the year, with an expected prevalence of pregnancy malaria of about 20% in Southern Ghana [1]. The minimum sample size to assess differences by malaria parasitemia status was estimated at 243 (Epi Info 3.4, 95% confidence level). To compensate for losses to follow-up three hundred and twenty (320) pregnant women were enrolled.

Pregnant women living in Madina and its environs making initial attendance to seek antenatal care at the maternity unit of the AMC for the current pregnancy who had not received IPTp and planned to deliver at the AMC were enrolled for the study from July to August 2008 and followed to delivery. The infants were also followed at monthly intervals at the Child Healthcare Clinics for six months.

### Ethical considerations

Ethical clearance was received from the Institutional Review Board (IRB) of the Noguchi Memorial Institute for Medical Research (NMIMR), College of Health Sciences, University of Ghana, Legon. All participants provided informed consent prior to data collection.

### Procedures

Demographic survey details as well as information on knowledge, possession and use of Insecticide Treated Nets (ITNs) were collected using a structured questionnaire. Venous blood was collected from each participant into a 5ml ethylene diamine tetra-acetic acid (K3EDTA) vacutainer tubes (Becton Dickinson vacutainer systems, UK). Each tube was labelled with the participant's unique identification number and was stored in a cold box, which was immediately transported to the laboratories of the NMIMR. Haematological, parasitological and clinical chemistry analyses including the Glucose-6-phosphate dehydrogenase (G6PD) deficiency test were run for each blood sample. An automatic blood analyzer (sysmex, Germany) was used to obtain the haemoglobin concentration.

To determine asexual *P. Falciparum* malaria parasitaemia, two sets of thick and thin blood films per blood sample were prepared on glass slides and allowed to dry on a rack. The thin films were fixed in absolute methanol and stained with 2% giemsa (BDH Laboratory Supplies Poole bh15 ITD, England) for ten minutes. The slides were then washed, left to dry and later examined in immersion oil under the microscope (Olympus BH2 Microscope, Japan) at X6 eyepiece and X100 oil immersion objective lens [14].The number of parasites per microlitre of blood in a thick film was determined in comparison with a standard number of leukocytes as described in the WHO Basic Malaria microscopy. Part I. Learner's guide [15].The asexual forms and gametocytes of P. falciparum were counted and recorded separately. All the slides were double-checked blindly by an independent microscopist for quality control. Placental blood smears were collected at delivery onto labelled microscopic glass slides. The slides were air dried and transferred to the laboratory for staining and examination of malaria parasites.

Infants were weighed at delivery using a previously calibrated scale at the labour ward and weights recorded on record sheets provided with the mother's unique identification number. The infants born to study participants were followed at monthly intervals for six months. The infants’ body temperature was determined at each visit.

Eligible women received Intermittent Preventive Treatment (IPTp) for malaria from hospital staff by direct observed therapy (DOT) at monthly intervals. Information about IPTp administration was initially entered by the hospital staff into the ANC attendance record booklets and was transferred onto the record forms provided for the study.

### Data analysis

The mean values for various parameters such as haemoglobin levels, and gestation were compared for the various categories of the pregnant women using standard statistical methods. Pair wise and three-way comparisons of mean Hb, proportion of low birthweight and prevalence of parasitaemia and parasite densities were done by Students't-test, ANOVA, and Fishers’ test. A value p< 0.05 was considered significant.

## Results

The characteristics of the pregnant women are shown as Table 1.

**Table 1:** Characteristics of pregnant women (n=320) attending ANC at Madina, Accra

Characteristic	Mean	95% Confidence interval
**Age (Years)**		
Age of pregnant women	28.42	27.8-29.04
Age at first pregnancy	23.95	23.03-24.88
**Gestation (weeks)**		
Gestation at registration	18.5	17.12-19.05
Gestation at first dose IPTp	23.53	22.91-24.15
**Level of Parasitaemia**		
Parasite density (µl blood)	1990.80	213.53-4195.13
**Haemoglobin concentration (g/dL) at registration**		
All pregnant women	11.44	11.29-11.8
Parasitaemic	10.74	9.79-11.67
Non parasitaemic	11.45	11.29-11.61
Primigravidae	11.38	11.08-11.67
Secundigravidae	11.56	10.91-12.21
Multigravidae	11.40	11.20-11.60

A significant proportion (46.6%) of participants lived outside Madina but within a 20 kilometer radius. Gestation at registration or first booking ranged from 2 weeks to as late as 39 weeks with a mean of 18.5 weeks (95% CI: 17.12 – 19.05). The prevalence of peripheral blood asexual P. falciparum parasitaemia on enrolment was 5% (n=15).

The mean haemoglobin level at registration for all the women was 11.44 g/dL (95% CI 11.29 – 11.80). Thirty-two percent of women had mild to moderate anaemia based on Hb<11g/dL as a cut-off. The lowest Hb recorded was 8.4g/dL among two women (Table 1) The mean Hb level of women who were diagnosed with peripheral blood parasitaemia (10.73g/dL) was significantly lower than that among women without parasitaemia (11.45g/dL). The mean Hb levels at first ANC visit did not differ significantly by parity (Table 1). Eleven percent (n=35) of women were found to have G6PD deficiency (data not shown).

Three percent of babies were of low birth weight. There was no significant association between the weight of babies and the number if IPTp doses received. None of the women who delivered infants with LBW had peripheral parasitaemia or anaemia at the time of first ANC registration but were among those who did not receive any IPTp during pregnancy. Microscopic examinations of the placental smears showed a 2.5% (n=3) prevalence of placental malaria at delivery. There was no association between placental parasitaemia and the incidence of LBW.

Knowledge of bed nets was high (97.3%). Sixty one percent of the women had ever slept in a net, however only 5% had slept under an ITN the night preceding the interview. ITN coverage was 31.6%. Fifty-five percent of women received IPT during pregnancy. Of those who received IPTp, 38% received three doses (Table 2).

**Table 2:** Summary results of use of ITNs and IPTp among pregnant women (n=320) attending ANC in Madina, Accra

Variable	Proportion
**Gravid status**	
Primigravidae	31.2%
Secundigravidae	3.7%
Multigravidae	64.0%
Unknown gravid status	0.6%
**ITNs**	
Awareness	97.3%
Ever slept in a net	60.5%
Have a bed net (treated + non treated)	41.2%
Have a treated net during pregnancy	31.6%
Slept in treated net the previous night	5.4%
**IPTp**	
Received IPT during pregnancy	55.0%
**IPTp doses (n=175)**	
First IPTp dose during 1st trimester	1.1%
First IPTp dose during the second trimester	74.8%
First IPTp dose during third trimester	24.0%
One dose of IPTp	28.0%
Two doses of IPTP	33.7%
Three doses of IPT	38.3%
**Birth-weight (n=121)**	
Birthweight<2.5 kg	3.3%
Birthweight ≥2.5kg	96.7%

## Discussion

The present study examined the prevalence of pregnancy associated malaria and its associated consequences including maternal anaemia and the incidence of low birth weight of infants and placental malaria among a group of pregnant women in a low malaria transmission setting in coastal Ghana. The prevalence of malaria parasitaemia on enrolment was much lower than what is reported in other studies in Ghana [1, 11].

Although, the absence of peripheral blood malaria parasites might reflect low rates of transmission of malaria in the study area (as indicated by relatively high Hb levels) or wide spread use of antimalarials as a result of the improved education on malaria during pregnancy in Ghana, in general, it is plausible that many women may have had submicroscopic peripheral blood parasites undetected by microscopy but detectable by the more sensitive PCR method [16, 17]. The low prevalence of peripheral blood parasitaemia may also have stemmed from the general affinity of the placenta to accumulate P. falciparum parasites [18–22] as was observed in 2.5% of the women. In addition, G6PD deficiency was higher among women in this study compared with levels observed in a cross-sectional study among pregnant women in the Ashanti region of Ghana [23]. Red blood cells with low G6PD activity offer a hostile environment to parasite growth and are thus protective for malaria infection. However, this protective effect also may lead to haemolysis from sulphonamides including antimalarials [24] like sulphadoxine pyrimethamine (SP) used in IPTp. With a relatively high prevalence of G6PD deficiency in Madina, testing for G6PD should be an important pre-requisite for antimalarial treatment including IPTp.

Pregnant women with anaemia may have an increased risk for poor pregnancy outcomes, particularly if they are anaemic in the first trimester. Although women in the present study had a relatively low prevalence of anaemia compared with other studies in Ghana [1, 11] almost one in three women had mild to moderate anaemia and women with P. falciparum infection had lower haemoglobin levels. These findings highlight the need for policies aimed at treating anaemia and limiting malaria infection in pregnant women in Madina.

Low birth weight attributable to malaria is associated with infant mortality. Only 3% of infants in this study had low birth weight. IPTp has been shown to improve birth weight [25, 26] hence most women in the study who were on IPTp had infants with birth weight equal or greater than 2500 kilograms.

Although malaria transmission in Madina is low to moderate, use of ITN was low despite high awareness levels. Low utilization of insecticide-treated nets was also observed in a malaria endemic area in Burkina Faso [27] where usage was limited due in part to community perceptions of the effectiveness of ITNs. The low usage may account for the parasitaemia detected albeit very low, in spite of IPTp administration during pregnancy [17]. These results suggest the need for public health education to encourage both the ownership and use of bed nets among pregnant women.

Some limitations must be acknowledged. First, helminthiasis and nutritional deficiencies are important causes of maternal anaemia in pregnancy. These factors were not investigated in the present study. Second, we were unable to examine the association between malaria parasitaemia and maternal anaemia and birth weight due to the small number of women who tested positive for P. falciparum infection. Third, although malaria transmission is seasonal with a peak during the rainy season (July to September), the very low prevalence of malaria among the pregnant women limited our ability to assess seasonality effects.

## Conclusion

The malaria prevalence on enrolment among the pregnant women was very low and was mostly asymptomatic. None of the women who delivered infants with LBW had peripheral parasitaemia or anaemia at the time of first ANC registration in their second to third trimesters. A high coverage of IPTp showed a reduced risk in maternal anaemia and LBW. Post partum studies need to be carried out on women who take IPTp. Although three of the women in the present study with G6PD deficiency had malaria infection at first presentation at ANC, the number was too low to make any meaningful conclusion about its protective effect. High knowledge of ITNs has not translated into use and even the few women who had ITNs were not sleeping in them. The non-use of ITNs has implications for malaria infection. The absence of clinical symptoms in the infants does not rule out infection especially with the low rate of use of ITNs.
